# Aircraft Engine Fault Diagnosis Model Based on 1DCNN-BiLSTM with CBAM

**DOI:** 10.3390/s24030780

**Published:** 2024-01-25

**Authors:** Jiaju Wu, Linggang Kong, Shijia Kang, Hongfu Zuo, Yonghui Yang, Zheng Cheng

**Affiliations:** 1 Institute of Computer Application China Academy of Engineering Physics, Mianyang 621999, China; 2 College of Civil Aviation, Nanjing University of Aeronautics and Astronautics, Nanjing 210016, China

**Keywords:** aircraft engine, fault diagnosis, 1DCNN, BiLSTM, attention mechanism

## Abstract

As the operational status of aircraft engines evolves, their fault modes also undergo changes. In response to the operational degradation trend of aircraft engines, this paper proposes an aircraft engine fault diagnosis model based on 1DCNN-BiLSTM with CBAM. The model can be directly applied to raw monitoring data without the need for additional algorithms to extract fault degradation features. It fully leverages the advantages of 1DCNN in extracting local features along the spatial dimension and incorporates CBAM, a channel and spatial attention mechanism. CBAM could assign higher weights to features relevant to fault categories and make the model pay more attention to them. Subsequently, it utilizes BiLSTM to handle nonlinear time feature sequences and bidirectional contextual feature information. Finally, experimental validation is conducted on the publicly available CMAPSS dataset from NASA, categorizing fault modes into three types: faultless, HPC fault (the single fault), and HPC&Fan fault (the mixed fault). Comparative analysis with other models reveals that the proposed model has a higher classification accuracy, which is of practical significance in improving the reliability of aircraft engine operations and for Remaining Useful Life (RUL) prediction.

## 1. Introduction

As one of the core components in the aerospace field, the health condition of aircraft engines significantly impacts the stable and reliable operation of aircrafts. With the continuous development of the aviation industry and an increasing demand for engine performance, the internal structure of aircraft engines has become increasingly complex. Many precision components of the engine need to operate reliably under harsh conditions such as high temperature, high pressure, high speed, and high load during routine flights. This results in a higher frequency of component failures. Additionally, due to varying flight scenarios and durations, the different components of aircraft engines may experience different levels of performance degradation, leading to various types of faults.

To reduce unnecessary maintenance efforts and associated human, material, and financial costs, as well as to promote the digital development of the aviation industry, precise maintenance and condition-based maintenance have become crucial goals in the field of aviation maintenance. Prognostic and Health Management (PHM) technology for equipment fault prediction and health management is considered an effective means to ensure operational quality, improve operational efficiency, reduce resource consumption, lower maintenance costs, and ensure reliable equipment operation [1,2]. PHM for aircraft engines plays a crucial role in providing intelligent maintenance solutions and preventing catastrophic accidents. Therefore, the fault diagnosis of aircraft engines, as an essential component of PHM, holds paramount importance.

Currently, fault diagnosis methods for equipment such as aircraft engines can be broadly categorized into three types: physics model-based methods, data-driven methods, and hybrid model-based methods combining both physics models and data-driven models. Physics-based modeling methods utilize real physical models and the digital simulations of equipment to establish degradation models, assess current health status, and predict future conditions. For instance, Im et al. proposed a model-based online fault diagnosis method that estimates fault severity indices based on negative sequence currents, identifying inter-turn faults in induction motors [3]. However, physics-based modeling methods face challenges in obtaining universally applicable physical simulation models, especially with the increasing complexity and diversity of physical entities. These methods often require substantial prior knowledge and expert experience, limiting the generalizability of physical simulation models.

With the rapid development of the Internet of Things (IoT) and the acceleration of industrial digital transformation, data-driven fault diagnosis methods have gained prominence. These methods leverage big data technologies, combining data mining and artificial intelligence (AI) techniques, to directly learn the behavior features of equipment from sensor monitoring data. Wang et al. proposed an innovative method called MTF-CNN, which automatically learns data features by combining Markov Transition Fields (MTF) and Convolutional Neural Networks (CNN) for the fault diagnosis of rolling bearings [4]. Data-driven methods do not require an in-depth understanding of the complex internal workings of the equipment. By collecting abundant degradation data from physical entities, these methods learn fault degradation features and classify fault modes, making them the mainstream research direction currently. Hybrid methods, combining both physics and data-driven approaches, aim to address the interpretability issues often associated with purely data-driven models. Zhou et al. introduced a Data-Model Cooperative Linking framework based on end-to-end deep network sparse denoising, utilizing both data- and model-driven elements for conducting fault diagnosis [5]. However, the application threshold of hybrid methods is higher, often requiring profound expert knowledge and experience in physical entities.

With the continuous development of IoT technology, edge devices generate increasingly abundant usable data. Data-driven methods based on big data technologies offer advantages of simplicity, efficiency, and convenience, garnering widespread attention from academia and industry. Particularly with the assistance of computing power and intelligent chips, AI technologies can be deployed on edge devices, enabling the application of deep learning algorithms such as Convolutional Neural Networks (CNNs), Recurrent Neural Networks (RNNs), Stacked Denoising Autoencoders (SDAEs), and Deep Belief Networks (DBNs) in practical industrial production and management. Compared to traditional shallow machine learning models, deep learning algorithms could model nonlinear systems and automatically learn intrinsic degradation features from data. Deep learning models to some extent mitigate individual differences between monitoring data, reduce the impact of data noise, and address issues such as insufficient generalization and susceptibility to local optima associated with traditional methods. For example, Wen et al. proposed a hybrid fault diagnosis method using ReliefF-Principal Component Analysis (PCA) and DNN, achieving an improved accuracy for wind turbine fault diagnosis [6]. Additionally, edge equipment, producing its usable data, has led to a surge in the application of data-driven methods based on big data technologies due to their simplicity, efficiency, and convenience. Notably, deep learning algorithms, including 1DCNN, have demonstrated excellent performance in handling time-dependent sequential data.

One-dimensional Convolutional Neural Network (1DCNN) has been employed in recent years to process time-dependent sequential data, showing a good performance in accuracy and efficiency. Raw data can be directly input into the model for training, allowing the exploration of temporal dependencies between data and addressing the limitations of previous models. Wang et al. proposed a novel method that combines features from multiple sensors using 1DCNN for predicting bearing faults [7]. Du X et al. proposed a fault diagnosis method for rotating machinery using a sequence Transformer model based on SPBO-SDAE and attention mechanism. Firstly, the Student Psychology Based Optimization Algorithm (SPBO) is used to adaptively select hyperparameters for the SDAE network. Then, the SPBO-SDAE network is used to extract the features of the original high-dimensional data layer by layer. This method has significant advantages in generalization performance, fault diagnosis accuracy, and time efficiency [8]. Jiao J et al. proposed a fault diagnosis method based on DBN joint information fusion technology. Firstly, wavelet transform is used to denoise, decompose, and reconstruct the vibration signals of industrial robot joint bearings. Then, a normalized feature vector is established for reconstructing energy entropy, and the normalized feature vector is used as an input for DBN. Finally, a combination of DBN and wavelet energy entropy technology is used for the fault diagnosis of industrial robots [9]. However, most of these studies require other algorithms to assist in feature extraction, or to use other algorithms to convert the original signal into an image before conducting fault diagnosis. While obtaining the fault diagnosis model, a significant amount of time was also spent on feature extraction and signal conversion. In recent years, 1DCNN has been used to process temporal data with time-dependent characteristics, demonstrating a good performance in terms of accuracy and time. At the same time, the raw data can be directly input into the model for training, which not only mines the temporal dependencies between data but also solves the shortcomings of the above model. Wang X et al. proposed a new method for fusing multimodal sensor signals. Firstly, features are extracted from the original vibration signal and acoustic signal, and then a deep neural network based on 1DCNN is used for fusion to predict bearing faults [10]. Yaser Ali Almatheel et al. proposed a 1DCNN with two convolutional layers to diagnose the time-domain vibration signals of bearings. Combining dataset augmentation techniques, 1DCNN will directly act on time-domain vibration signals to diagnose bearing faults [11]. Wang Y et al. proposed a bearing fault feature extraction and recognition method based on Particle Swarm Optimization for Maximum Related Kurtosis Deconvolution (MCKD) and 1DCNN. Firstly, fault feature selection is performed on the multi-channel signals of rolling bearings, and signals containing fault features are filtered using MCKD. Then, 1DCNN is used to identify faults in feature signals under different damage conditions [12]. Zhang S et al. proposed a novel fault detection method using Zero Shot Learning (ZSL). This method first extracts features from the original signal by applying 1DCNN, then establishes semantic descriptions as shared fault attributes between known and unseen faults, and finally uses bilinear compatibility functions to find the highest level of bearing fault type [13].

In the process of equipment fault degradation, signals collected by sensors change over time. 1DCNN alone may not capture the bidirectional temporal dependencies between data. Furthermore, there is limited literature that investigates fault diagnosis on the CMAPSS dataset. Han et al. proposed a 1DCNN-based aircraft engine fault mode classification model under multiple operating conditions. They clustered six flight conditions and classified fault modes into two categories: HPC fault and HPC&Fan mixed fault. They then established a 1DCNN binary classification fault diagnosis model [14]. However, this approach did not consider bidirectional temporal dependencies between data and did not diagnose the fault mode of aircraft engines with the faultless mode. During the initial flight cycles of aircraft operations, the engine operates in a healthy mode (i.e., faultless). Considering all fault modes that aircraft engines may experience throughout their entire life cycle, this paper proposes the aircraft engine fault diagnosis model based on 1DCNN-BiLSTM with CBAM. This model does not require the integration of other feature extraction algorithms or the conversion of sensor signals into time–frequency images. To train the model in a supervised manner, labels are assigned to data for the faultless mode, the HPC fault (single-fault) mode, and the HPC&Fan fault (mixed-fault) mode. Additionally, as monitoring values from different components in different flight scenarios may have significant dimensional differences, the original sensor data is standardized in this study. Finally, the preprocessed data is input into the 1DCNN-BiLSTM model with CBAM for training to obtain the fault diagnosis model. The major innovative points in this article are summarized as follows:

(1) The proposed model can be directly applied to raw monitoring data without the need for additional algorithms to extract features.
(2) A channel and spatial attention mechanism (CBAM) is added after the 1DCNN layers, which could assign higher weights to features relevant to fault categories and make the model pay more attention to them. Also, BiLSTM is added after the CBAM layer, capturing the nonlinear time feature sequences and bidirectional contextual feature information, to improve the prediction accuracy and model performance.
(3) In addition to diagnosing various faulted modes of aircraft engines, the faultless mode is also diagnosed, which has played a positive role in further predicting the RUL and spare parts management.

The content arrangement of this paper is as follows: Section 2 provides a detailed introduction to the fault diagnosis framework for aircraft engines. Section 3 presents experimental verification results on the CMAPSS dataset. Section 4 summarizes the paper, addressing some limitations and prospects.

## 2. Fault Diagnosis Model Based on 1DCNN-BiLSTM with CBAM

### 2.1. 1DCNN

**Figure 1:**
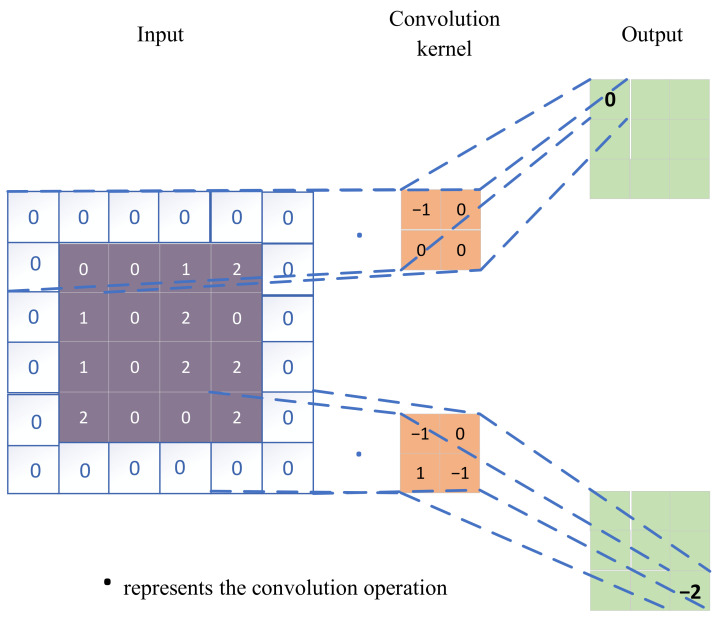
Convolutional operation structure diagram.

Convolutional Neural Networks (CNN) have their roots in the concept of visual receptive fields proposed in the 1960s, with formal development initiated by Yann Lecun of New York University in 1988. CNN has consistently demonstrated outstanding performance in the field of image processing. Deep network architectures based on CNN, such as LeNet-5, AlexNet, VGGNet, and ResNet, have earned prestigious positions in the top conferences focusing on image processing. In comparison to other neural network structures, CNN’s most distinctive feature lies in the incorporation of convolutional layers and pooling layers. The mathematical expression and simplified structure diagram for a single convolution operation are presented in Equation (1) and Figure 1:

(1) $$y_{c}=f_{c}\left(x\cdot k_{c}+b\right)$$

Here, $f_{c}$ represents the activation function, x denotes the input, · represents the convolution operation, $k_{c}$ represents the convolutional kernel, and b is the bias term.

**Figure 2:**
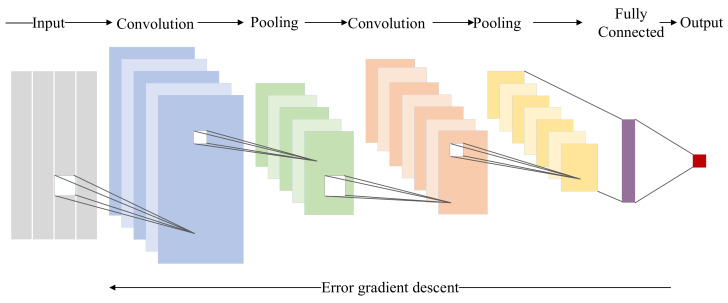
Deep network architecture of CNN.

1DCNN are specifically designed for processing time series data, distinguishing them from CNNs employed in image processing. In 1DCNN, the convolutional kernels are one-dimensional. Figure 2 illustrates a deep network architecture based on 1DCNN tailored for addressing time series problems.

**Figure 3:**
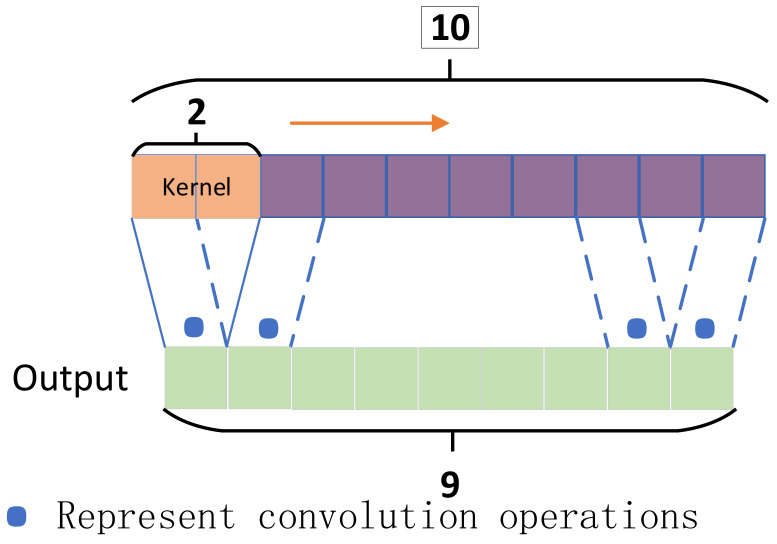
One-dimensional convolution operation of 1DCNN.

In the context of equipment fault diagnosis and maintenance assurance, the convolutional kernel, in essence, represents a weight matrix. As the kernel slides, it sequentially performs local convolution operations on one-dimensional input data along the time series. The schematic diagram of one-dimensional convolution is illustrated in Figure 3. Assuming the one-dimensional input data has a dimension of ten, with a kernel size of two and a stride of one, the convolutional kernel slides along the direction indicated by the orange arrow, performing convolution operations on the data. The final output dimension is nine. The purpose of convolution is to extract deep features from the input. Following convolution, a pooling layer is employed to compress the data and parameter quantities. The key characteristics of CNN models are local connectivity and weight sharing. By reducing the number of weights, the complexity of the network model is diminished, simplifying the training and optimization processes and, to a certain extent, mitigating the risk of model overfitting.

Compared to other temporal models such as RNN, 1DCNN exhibits a faster convergence during training. In recent years, it has demonstrated model performance in equipment component fault diagnosis and Remaining Useful Life prediction fields that are comparable to LSTM. In reference [15], a generative adversarial network was employed for data augmentation on bearing data, followed by adversarial training using 1DCNN on the augmented data. This approach resulted in a smaller average absolute deviation and mean square error for the RUL prediction.

### 2.2. Attention Mechanism

**Figure 4:**
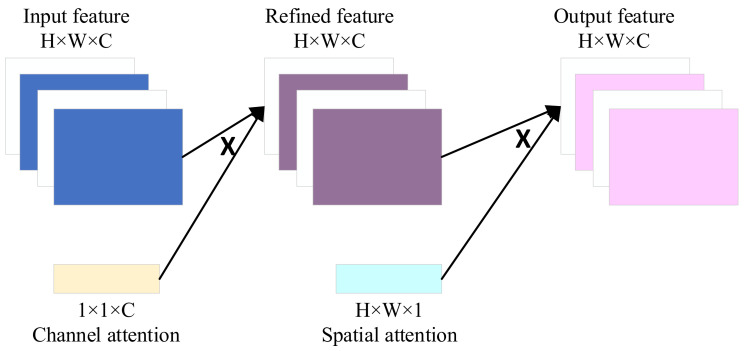
CBAM structure.

In the field of deep learning, models often need to receive and process a large amount of high-dimensional data. However, after feature extraction, the 1DCNN assumes that each fault feature channel is equally important during the convolutional pooling process, but in reality, the importance of information carried by each fault feature is different. In this case, an attention mechanism (AM) is needed to assign higher weights to the important features, making deep learning models more focused on these features. Convolutional Block Attention Module (CBAM), a simple yet effective attention module for feed-forward Convolutional Neural Networks, was first proposed by Sanghyun Woo [16] in 2018. CBAM consists of two independent sub-modules: channel attention module and spatial attention module. The structure of CBAM is shown in Figure 4.

**Figure 5:**
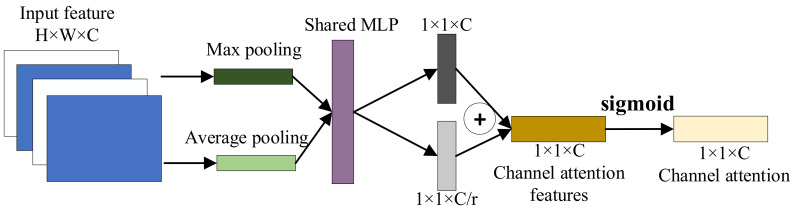
Channel attention module.

The channel attention module obtains corresponding fault features from the input fault features through average pooling and maximum pooling. Then, multiple perception layers are input to obtain two fault feature vectors. Two fault feature vector elements are added one-by-one and activated to obtain the corresponding fault channel attention features. The processing flow of the channel attention module is shown in Figure 5.

**Figure 6:**
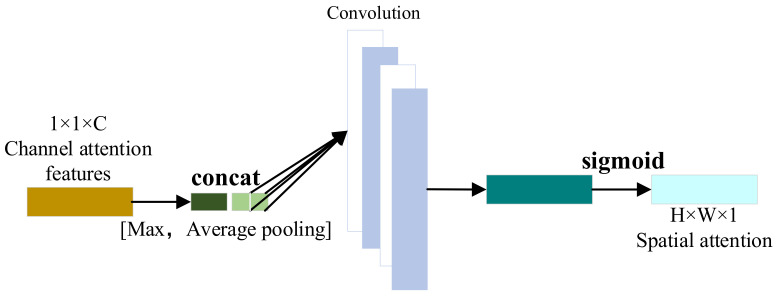
Spatial attention module.

The spatial attention module performs average pooling and maximum pooling operations on the aforementioned fault channel attention features to obtain two fault features. After concatenating the two fault feature channels mentioned above, the required features are obtained by multiplying them with the input fault features through convolution and activation operations. The processing flow of the spatial attention module is shown in Figure 6.

### 2.3. BiLSTM

The research on Recurrent Neural Networks (RNN) began in the early 1980s, representing a type of network designed for handling sequential data. As theoretical understanding and computational capabilities advanced, RNN evolved into a form of deep learning algorithm in the early 2000s and has been extensively applied in the field of Natural Language Processing (NLP). The essence of RNN lies in its ability to retain memory, enabling it to remember past sequential information and capture relationships between data at different time steps. As the health status of equipment components degrades over time, data monitored by sensors is inherently interconnected.

**Figure 7:**
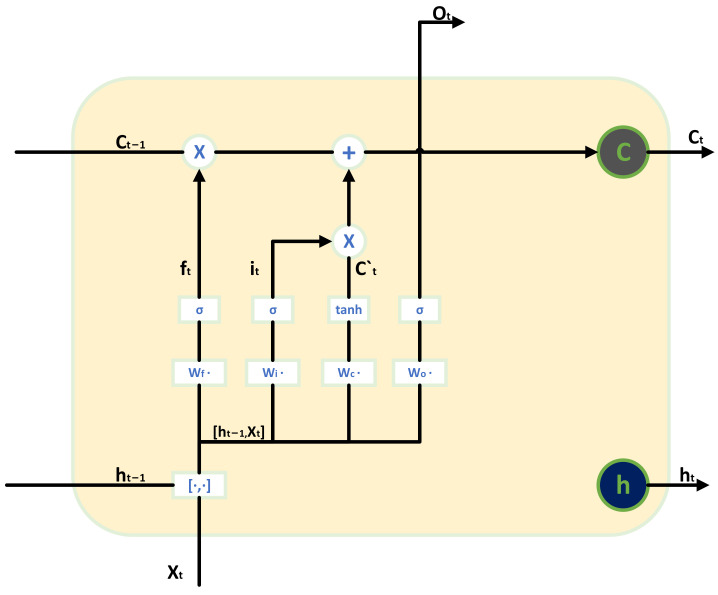
LSTM internal door structure.

However, RNN encounters challenges during training, such as the issue of long-term dependencies among data and the potential for exploding or vanishing gradients during the backpropagation of errors. Long Short-Term Memory (LSTM) networks, a specialized form of RNN, address these challenges by mitigating the vanishing gradient problem and handling long-term dependencies in time series data. The internal structure of LSTM is depicted in Figure 7.

As can be seen from Figure 7, the LSTM structure has three controlled gates, which are called the forget gate, the input gate, and the output gate. The forget gate processes the information of $h_{t-1}$ and $x_{t}$ through the sigmoid cell to determine how much information is retained by the cell state $C_{t-1}$, as shown in Equation (2).

(2) $$f_{t}=\sigma \left(W_{f}\cdot \left[h_{t-1},x_{t}\right]+b_{f}\right)$$

(3) $$\sigma \left(x\right)=\frac{1}{1+e^{-x}}$$

The input gate processes the information of $h_{t-1}$ and $x_{t}$ through the sigmoid unit to determine how much information to update. The updated information is obtained by processing the information of $h_{t-1}$ and $x_{t}$ by the tanh unit, as shown in the following equations:

(4) $$i_{t}=\sigma \left(W_{i}\cdot \left[h_{t-1},x_{t}\right]+b_{i}\right)$$

(5) $${\tilde{C}}_{t}=\tanh\left(W_{C}\cdot \left[h_{t-1},x_{t}\right]+b_{C}\right)$$

(6) $$\tanh\left(x\right)=\frac{e^{x}-e^{-x}}{e^{x}+e^{-x}}$$

Then, the cell state $C_{t-1}$ is updated to $C_{t}$ according to the input gate and the forget gate, as shown in the following Equation (7):

(7) $$C_{t}=f_{t}*C_{t-1}+i_{t}*{\tilde{C}}_{t}$$

Finally, the final $h_{t}$ is determined according to the cell state $C_{t}$, the output gate, $h_{t-1},$ and the input $x_{t}$, as shown in the following Formulas (8) and (9):

(8) $$o_{t}=\sigma \left(W_{o}\cdot \left[h_{t-1},x_{t}\right]+b_{o}\right)$$

(9) $$h_{t}=o_{t}*\tanh\left(C_{t}\right)$$

Here, ∗ represents the Hadamard product. The output $h_{t}$ is then fed into the next layer to continue the aforementioned operations. In reference [17], a fault diagnosis method for ship power plants based on LSTM is proposed, addressing the long-term dependency issue of RNN, demonstrating high interpretability, and obtaining high accuracy and precision in the testing dataset.

**Figure 8:**
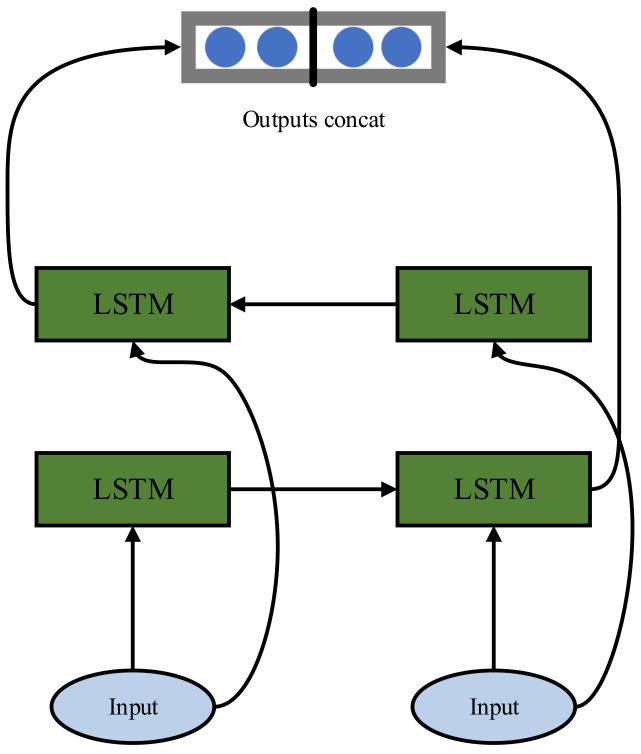
Single-layer BiLSTM structure.

However, LSTM can only capture forward dependencies, whereas Bidirectional LSTM (BiLSTM) can better capture both forward and backward dependencies between data. A single layer of BiLSTM is composed of two LSTMs: one processes the input sequence from left to right, and the other processes it from right to left. After completion, the outputs of the two LSTMs are concatenated and passed to the next layer of the network. The network structure is depicted in Figure 8.

In Figure 8, only after all time steps in both forward and backward directions are computed, the results from these two directions are concatenated to obtain the final BiLSTM output. In reference [18], an interpretable bearing fault diagnosis model based on CNN-BiLSTM is proposed. This model demonstrates a high diagnostic accuracy by leveraging CNN for feature extraction and analysis, followed by fault diagnosis with BiLSTM.

### 2.4. Fault Diagnosis Framework

**Figure 9:**
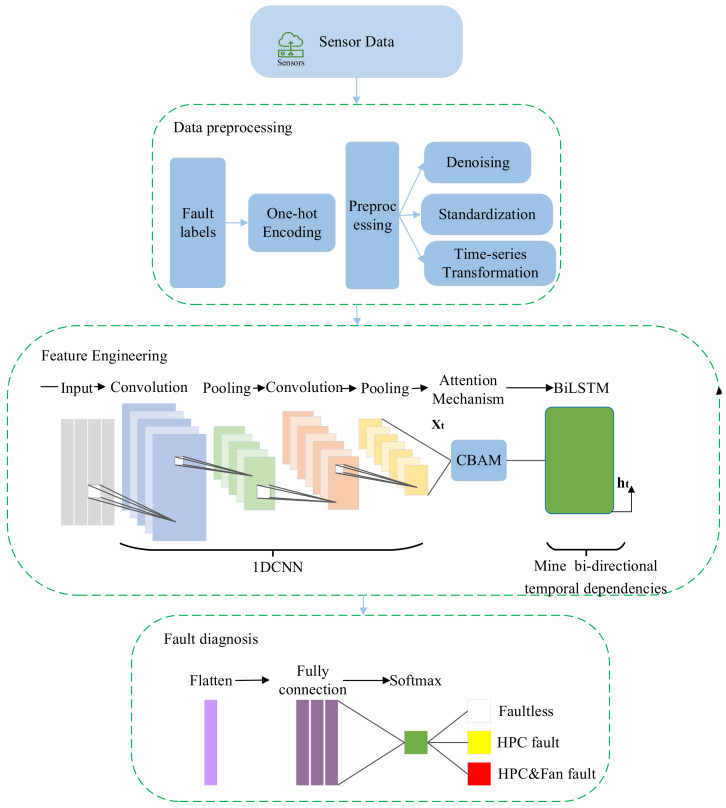
Fault diagnosis model based on 1DCNN-Bilstm with CBAM.

Usually, in the process of training neural networks, in order to improve the model’s ability and performance to fit nonlinear systems, it is possible to increase the number of layers in the network or the number of neurons in each layer. But as the number of network layers and neurons increases, the number of network parameters that need to be trained will also increase. The weight sharing and local connections of CNN class models can reduce the weight parameters that need to be trained in the network and extract spatial features. The attention mechanism can assign different weights to features and improve the focus on important features. BiLSTM can mine simple bidirectional temporal dependencies of features and extract temporal features. This paper proposes the fault diagnosis model based on 1DCNN-BiLSTM with CBAM, diagnosing the fault modes generated throughout the entire life cycle of aircraft engines. This model combines feature extraction, feature weight optimization, bidirectional temporal dependency extraction, and fault mode classification tasks into one network model, achieving high prediction accuracy. It has practical value for improving the reliability of aircraft engine operation and further RUL prediction. And the proposed aircraft engine diagnosis model based on 1DCNN-BiLSTM with CBAM is shown in Figure 9.

From Figure 9, it can be seen that the proposed fault mode classification and diagnosis model can be divided into three parts, i.e., data preprocessing, feature engineering, and fault diagnosis, which are discussed in detail in the following Section 2.4.1, Section 2.4.2 and Section 2.4.3

#### 2.4.1. Data Preprocessing

The model proposed in this paper is a supervised learning model that requires labeling different fault modes. The fault modes are categorized into three classes: healthy data is referred to as the ‘faultless’ mode with a label of ‘0’; data with a single fault is termed the ‘single fault’ mode with a label of ‘1’; and data with two faults is termed the ‘mixed-fault’ mode with a label of ‘2’.

The processing of sensor data involves three main steps:

1. Noise Removal: Sensors with constant measurements with increasing flight cycles are eliminated. Sensors with constant values are considered to not affect the engine’s fault degradation process. Here, one flight cycle typically serves as the unit of engine operating time, representing a complete flight process from takeoff to landing.

2. Standardization: Due to different units of measurement and significant variations in sensor measurements across various flight scenarios, directly inputting this data into a neural network model may result in uneven feature weight distribution. This not only slows down the model’s learning and convergence speed but also leads to inaccurate prediction results. Therefore, before training the model, this paper normalizes the raw sensor data.

3. Time Series Transformation: The original sensor data is measured based on the flight cycle count of the aircraft, isolating the dependency relationships of each flight cycle. As the degradation of a single flight cycle is often closely related to the preceding flight cycles, the paper performs a time series transformation on the original sensor data.

#### 2.4.2. Feature Engineering

1. 1DCNN for Spatial Features

The 1DCNN can utilize convolutional kernels to automatically learn the internal relationships of sensor data in the sliding direction and extract effective features related to fault degradation. The feature extraction process of the 1DCNN model is as follows:

(1) Convolutional Layer: Preprocessed data is input into the 1DCNN model, and the convolutional kernel operates on the output features of the previous layer. The degradation features of fault information are obtained through an activation function. The one-dimensional convolutional operation is expressed in Equation (10):

(10) $$y_{j}^{l}=f\left[Bn\left(conv1D\left(\sum_{h\in M_{j}}y_{h}^{l-1}*\omega_{hj}^{l}+b_{j}^{l}\right)\right)\right]$$

where $y_{h}^{l-1}$ represents the h−th degradation feature of the (l−1)th layer, $y_{j}^{l}$ represents the j−th overflight feature of the l−th layer, $M_{j}$ represents the set of input features from the previous layer, $\omega_{hj}^{l}$ represents the weight matrix of the convolutional kernel, $b_{j}^{l}$ represents the bias, and Bn represents Batch Normalization, which aligns input data to the same distribution, thereby accelerating the convergence speed of the network. To some extent, it can also prevent the occurrence of gradient explosion or vanishing problems during the model training process. f[·] represents the activation function, and in this paper, Rectified Linear Unit (ReLU) is chosen as the activation function, expressed in Equation (11):

(11) f(x)=max(0,x)

(2) Pooling Layer: The pooling operation in the pooling layer compresses the dimension of features, reducing model parameters, improving computational efficiency during model training, and accelerating model convergence. This paper selects max-pooling as the pooling operation, calculated as in Equation (12):

(12) $$y_{j}^{l}=\underset{\left(j-1\right)H+1\leq k\leq jH}{max}y_{k}^{l-1}$$

where H represents the width of the convolutional kernel.

(3) After several layers of convolution and pooling operations, local spatial features related to fault degradation are obtained.

2. Attention Mechanism

CBAM dynamically selects local feature information from 1DCNN output, which can enhance fault information while suppressing noise and irrelevant information. By assigning different weights to features, the higher the importance of fault feature information, the greater the weight, while the lower the importance of fault feature information, the smaller the weight.

3. BiLSTM for Sequential Features

By adopting a bidirectional structure, the BiLSTM network processes input sequences in both forward and backward directions. This bidirectional nature enhances the network’s ability to capture information from past and future time steps, enabling a more comprehensive understanding of the temporal relationships within the data. In the context of fault diagnosis, this bidirectional capability is advantageous for capturing relevant features that may exhibit patterns in both directions of time.

#### 2.4.3. Fault Diagnosis

Using the bidirectional temporally enhanced features obtained in part 3 of Section 2.4.2 for equipment fault modes, this paper performs the fault mode classification employing a softmax classifier. The probability of the ith input sample being classified into category k is given by the following:

(13) $$p\left(y^{i}=k|x^{i};\theta \right)=\frac{e^{\theta_{k}^{T}x}}{\sum_{i=1}^{k}e^{\theta_{i}^{T}x}}$$

Here, $p\left(y^{i}=k|x^{i};\theta \right)$ denotes the probability of the ith input sample belonging to category k,$x^{i}$ represents the input features, $\theta_{k}$ is the weight vector for class k, and k is the total number of classes. The softmax function ensures a normalized probability distribution across all classes. During the model training process, the error gradient descent backpropagation algorithm is employed. The parameters of the proposed model are fine-tuned through supervised learning using one-hot fault labels.

## 3. Experimental Analysis

### 3.1. Dataset

This paper validates the effectiveness of the 1DCNN-BiLSTM with the CBAM model on the publicly available Commercial Modular Aero-Propulsion System Simulation (CMAPSS) dataset from the National Aeronautics and Space Administration (NASA). The dataset simulates the actual degradation process of a turbofan engine from the healthy state to the Run to Failure (RtF) overflight cycles. The dataset is divided into four different subsets, as shown in Table 1.

The engine’s fault modes consist of two types, where the single fault mode datasets FD001 and FD002 only include High-Pressure Compressor (HPC) degradation, and the mixed fault mode datasets FD003 and FD004 include HPC&Fan degradation. Additionally, there are two types of operating conditions: FD001 and FD003 include only a single operating condition, while FD002 and FD004 encompass multiple operating conditions. In each subset, training sets, test sets, and datasets recording the actual RUL labels are provided. The training and test sets include engine ID, operational cycle count, three operating condition parameters of the engine (flight height H, Mach number Ma, and throttle resolver angle TRA), and measurements from 21 sensors. The training set records the engine’s degradation process from a healthy state to states of increasing severity until the system’s Run to Failure (RtF).

**Figure 10:**
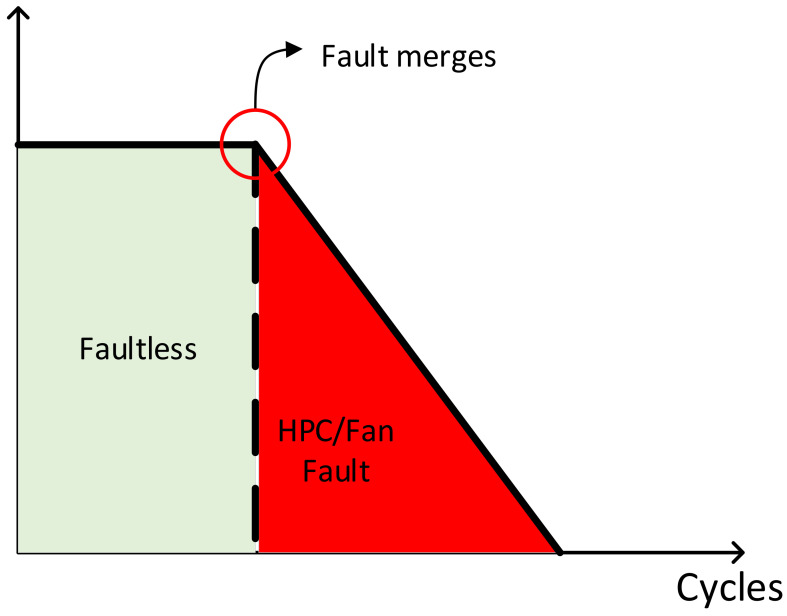
Fault piecewise linear degradation model.

Simultaneously, the CMAPSS dataset has been widely used for predicting the RUL of turbofan engines. In most of the literature on RUL prediction [19,20,21,22], the degradation of RUL is treated as a piecewise linear degradation model, as illustrated in Figure 10.

In Figure 10, for the initial several cycles, the RUL is assigned a fixed value. This fixed value is referred to as the early RUL. As the flight cycles continue to increase, when the RUL value reaches the early RUL, the RUL follows a linear degradation model. Therefore, during the early RUL period, the aircraft operates smoothly and healthily, and no faults are assumed to occur. In the linear degradation phase of RUL, the engine is considered to be in a faulty state. Based on this, this paper selects the FD001 and FD003 datasets. Following the research in the literature [19,20], the early RUL value is set to 125. The fault modes are categorized into three classes (faultless, HPC single fault, HPC&Fan mixed fault) for analysis.

### 3.2. Data Preprocessing

(1) Fault Label Encoding

This paper categorizes fault modes into three labels, i.e., faultless, HPC single fault, and HPC&Fan mixed fault, for the supervised training of the proposed model. To prevent any extraneous information interference from class variable values having no actual meaningful relationship to the model, it is necessary to perform one-hot encoding on these fault categories before training the model, as shown in Table 2. Faultless mode is encoded as [1,0,0], HPC single fault mode as [0,1,0], and HPC&Fan mixed fault mode as [0,0,1].

(2) Denoising

**Figure 11:**
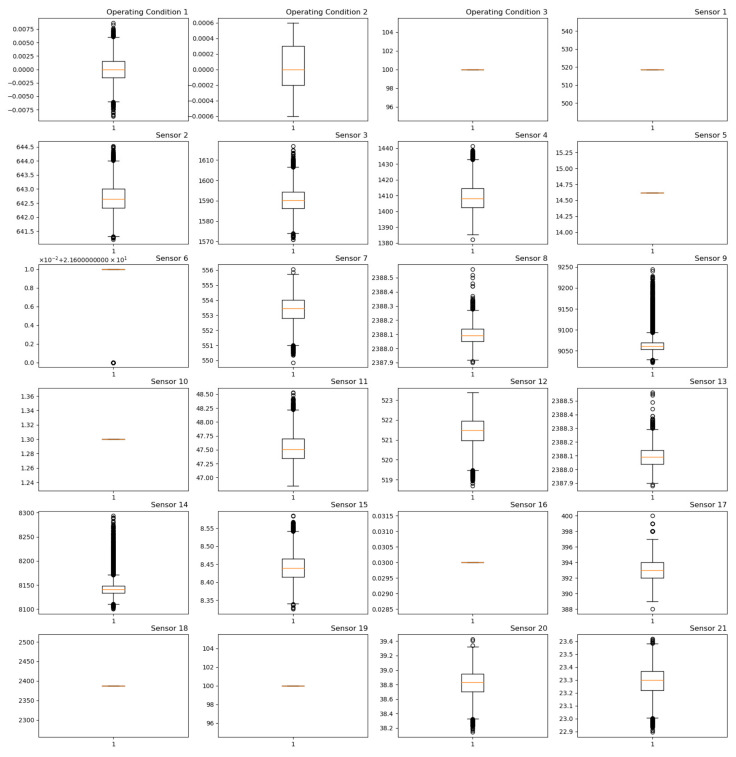
Box diagram of 3 operating conditions and 21 sensors.

As the aircraft continuously flies and lands, certain sensors exhibit constant readings. Throughout the engine’s entire lifecycle, these sensors can be considered irrelevant to the engine’s aging process and should be excluded. In order to depict the distribution of the original sensor data, this paper presents boxplots for the three operating condition parameters and 21 sensors, as shown in Figure 11.

From Figure 11, it is observed that the values of operational parameter 3 and sensors 1, 5, 6, 10, 16, 18, and 19 are constant and should be removed. Additionally, since the operating conditions for FD001 and FD003 are the same, the influence of operational parameters will not be considered, and only the measurements from sensors will be used. Therefore, this paper retains 14 sensors [19], namely, T24, T30, T50, P30, Nf, Nc, Ps30, Phi, NRf, NRc, BPR, htBleed, W31, and W32. The specific meanings of these sensor parameters are detailed in Table 3.

(3) Standardization

As observed from Figure 7, the dimensions of the monitored values for various sensors differ significantly. Directly inputting these data into the network model would result in uneven weight distribution for input features, making the model unable to converge. Therefore, this paper individually performs mean subtraction and variance normalization on the monitoring data of each sensor listed in Table 3, as shown in Formula (14).

(14) $$X^{*}=\frac{X-\mu }{\sigma }$$

where X represents the original sensor data, μ is the mean of all samples for one sensor, σ is the standard deviation of all samples for one sensor, and $X^{*}$ represents the normalized data. Data standardization accelerates the convergence of gradient descent to the optimal solution and improves the precision and accuracy of prediction results.

(4) Time Series Transformation

Following the time window selection in the literature, the time window size for the FD001 and FD003 datasets is set to 30 [19,22], with a stride of 1. The data scale before and after time series transformation for the FD001 and FD003 datasets is shown in Table 4.

After the time window transformation, the original unordered two-dimensional feature space is extended into the time dimension. Compared to the original data dimensions, the transformed data now has an additional time dimension. This helps the BiLSTM model in capturing temporal dependencies between features, aligning with the mechanism of fault degradation over time.

After the various steps of data preprocessing, we have tabulated the sample counts for the three fault modes, as shown in Table 5.

From Table 5, it can be observed that the proportions of each fault class to the total number of samples are relatively similar. The ‘Faultless’ mode has a larger number of samples, while the counts for ‘HPC Single Fault’ and ‘HPC&Fan Mixed Fault’ are nearly equal.

### 3.3. Evaluation Index

In order to verify and evaluate the effectiveness of the proposed method, this study applied four commonly used performance metrics: Macro-precision, Macro-recall, accuracy, and F-Score. Macro-precision represents the precision calculated for each class separately, followed by the arithmetic mean calculation with all categories. Macro-recall represents the recall calculated for each class separately, also followed by the arithmetic mean calculation with all categories. Accuracy represents the ratio of correctly classified samples to the total number of samples. The single category values of precision, recall, and accuracy can be obtained from the confusion matrix, as shown in Formulas (15)–(17):

(15) $$\mathrm{Precision}=\frac{TP}{TP+FP}$$

(16) $$\mathrm{Recall}=\frac{TP}{TP+FN}$$

(17) $$\mathrm{Accuracy}=\frac{TP+TN}{TP+TN+FP+FN}$$

Here, TP represents true positives, TN represents true negatives, FP represents false positives, and FN represents false negatives.

To comprehensively consider both Macro-precision and Macro-recall, the F-Score is introduced. It represents the weighted harmonic mean of Macro-precision and Macro-recall, as shown in Formula (18):

(18) $$F-\mathrm{Score}=\left(1+\beta^{2}\right)\frac{\mathrm{Precision}\times \mathrm{Recall}}{\beta^{2}\times \left(\mathrm{Precision}+\mathrm{Recal}\right)}$$

Here, *β* is commonly set to one, known as F1−Score, aiming to balance the impact of Precision and Recall on the prediction results and provide a relatively reasonable evaluation metric.

### 3.4. Result Analysis

This paper validated the proposed model on the ‘train_FD001’ and ‘train_FD003’ subsets of the CMAPSS dataset. The dataset was randomly split, allocating 70% of the data for training, 20% for validation, and 10% for testing. The parameters of the models were trained and updated on a personal computer equipped with Inter Core i5 processor, 2.5 GHz clock speed, and 16 GB RAM.

#### 3.4.1. Result Comparison

To verify the effectiveness of the proposed model, the results are compared with traditional shallow machine learning models, such as Support Vector Machine (SVM) [23,24,25,26] and Random Forest (RF) [25,27,28], and deep learning models: Fully Connected Neural Network (FNN) [29], Recurrent Neural Network (RNN) [30], 1DCNN [7,10,11], LSTM [17], and BiLSTM [18] models. The average accuracy, Macro-precision, Macro-recall, and F1-Score on the test set are shown in Table 6.

According to Table 6, the average accuracy, Macro-precision, Macro-recall, and F1-Score of SVM, FNN, and RNN are not significantly different. Due to the ability of RNN to extract temporal features of faults, the classification effect is better; RF is an integrated learning model, and its effect is better than that of single models such as SVM, FNN, and RNN, but it belongs to traditional machine learning models, so its effect is not as good as that of complex deep learning models such as LSTM; both LSTM and BiLSTM are the improvements of RNN, incorporating gate mechanisms to alleviate the problem of vanishing gradients, resulting in better performance than RNN; BiLSTM performs better than LSTM due to its ability to extract bidirectional dependencies of features; due to the inability of RNN-based deep learning models to extract spatial features, 1DCNN not only extracts spatial features but also one-dimensional temporal features to a certain extent, resulting in a better classification performance than BiLSTM; however, 1DCNN assigns the same weight to the extracted features and cannot extract the bidirectional temporal dependencies of the features. The proposed 1DCNN-BiLSTM with the CBAM model not only assigns higher weights to fault-related features through CBAM, but also uses BiLSTM to extract the bidirectional temporal dependencies of the features, resulting in the highest average accuracy, Macro-precision, Macro-recall, and F1-Score, as can be seen in bold. Therefore, the model proposed in this article is effective for the fault mode classification of aircraft engines and has a good diagnostic performance.

#### 3.4.2. Ablation Experiments

To further demonstrate the effectiveness of the 1DCNN-BiLSTM with the CBAM model, we conducted ablation experiments to demonstrate the role of CBAM and BiLSTM. The average accuracy, Macro-precision, Macro-recall, and F1-Score of 1DCNN-LSTM, 1DCNN-BiLSTM, 1DCNN-LSTM with CBAM, 1DCNN with CBAM, and 1DCNN-BiLSTM with CBAM were compared on the test set, as shown in Table 7. Among them, in all the experiments, 1DCNN, LSTM, and BiLSTM all used the same network structure.

According to Table 7, on the test set, the average accuracy, Macro-precision, Macro- recall, and F1-Score of 1DCNN-BiLSTM are higher than those of 1DCNN-LSTM, and those of 1DCNN-BiLSTM with CBAM are higher than those of 1DCNN with CBAM, indicating the effectiveness of BiLSTM in mining feature bidirectional temporal dependencies. The average accuracy, Macro-precision, Macro-recall, and F1-Score of 1DCNN-LSTM with CBAM(bold in Table 7) are all higher than those of 1DCNN-LSTM, and those of 1DCNN-BiLSTM with CBAM are also higher than those of 1DCNN-BiLSTM. This indicates that the addition of CBAM focuses more attention on the fault-related features, resulting in a better predictive performance of the model. This once again confirms that the performance of this model is superior to other models.

#### 3.4.3. Visualization

**Figure 12:**
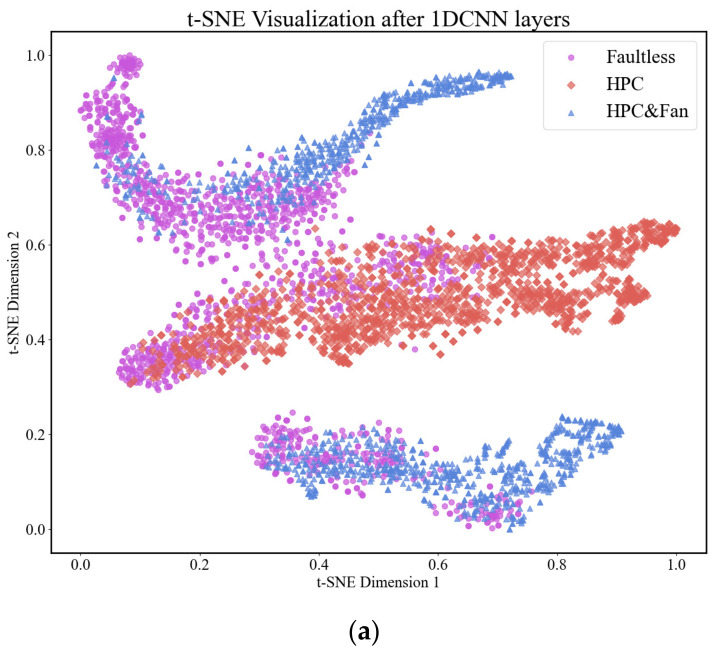

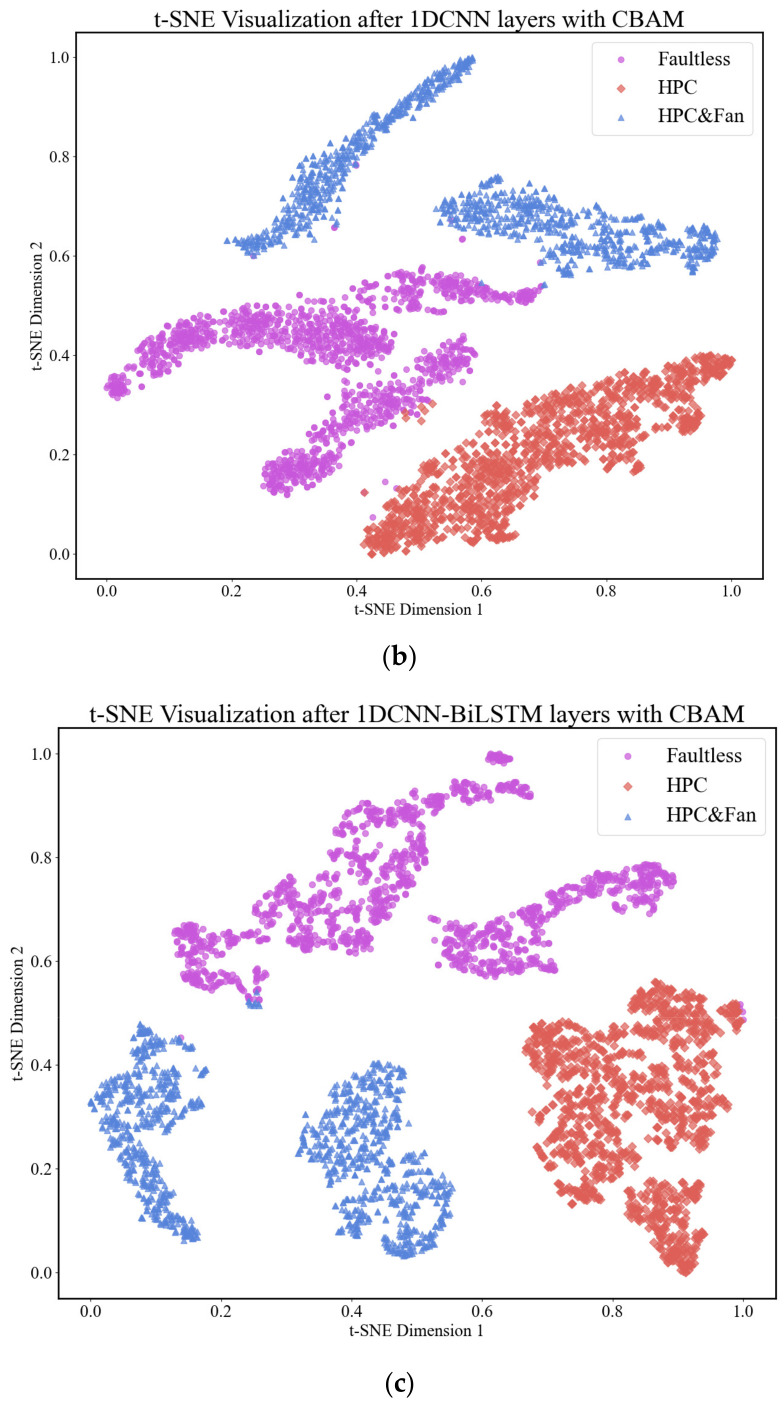
(**a**) The data distribution of feature only after the 1DCNN layers, (**b**) the data distribution of feature after the 1DCNN layers with CBAM, and (**c**) the data distribution of feature after the 1DCNN-BiLSTM layers with CBAM.

We also used t-SNE visualization technology to visualize the features of the model only with 1DCNN layers, 1DCNN layers with CBAM, and 1DCNN-BiLSTM layers with CBAM, as shown in Figure 12.

From the t-SNE visualization in Figure 12, it can be seen that the classification boundary in (b) is clearer than that in (a), but there are still some intersections. The distance between different classes in (c) is larger than that in (b), indicating that the network model with BiLSTM layer can cluster samples of the same category well and can also classify the samples of different categories well. The classification results are better than those for the network models only with 1DCNN layers and 1DCNN layers with CBAM, and this once again confirms that the performance of this model is more effective.

## 4. Conclusions

To address the issues of fault prediction and health management in aircraft engines, this paper proposed the aircraft engine fault diagnosis model based on 1DCNN-BiLSTM with CBAM. The publicly available CMAPSS dataset from NASA is used to validate the proposed model, classifying the aircraft engines into three fault modes (faultless, HPC single fault, HPC&Fan mixed fault). Through comparisons with other common diagnosis models, the proposed model demonstrated a higher classification accuracy. This is crucial for subsequent tasks such as RUL prediction, condition-based maintenance, and overall health management for aircraft engines.

In the future, with the continued development of digital twins and equipment health management, methods integrating real-time diagnosis and RUL prediction based on edge twin data and artificial intelligence will be deeply fused [31]. The proposed method in this paper provides a solid research foundation for such integration, offering valuable fault information to assist in predicting the RUL of equipment. However, there are still some limitations in this study:

(1) We only considered datasets with a single operating condition, without considering complex multi-operating condition problems in actual operating scenarios. In the future, we will use transfer learning [32,33] to expand the model to multi-operating condition backgrounds to enhance its robustness and applicability;
(2) There is no further detailed classification of categories with faults, as the process from the occurrence of faults to the complete failure is a long one. Therefore, in the future research, the diagnostic granularity of fault categories will continue to be refined, such as initial faults, moderate faults, and severe faults;
(3) In response to the demand for the implementation of artificial intelligence, we will conduct interpretability research on deep learning models in the future, in order to understand the ‘black box’ model on a semantic level in the actual industrial scenarios [34].

## Figures and Tables

**Table 1:** CMAPSS dataset.

CMAPSS	FD001	FD002	FD003	FD004
Engines in training set	100	260	100	249
Engines in test set	100	259	100	248
Operating conditions	1	6	1	6
Fault modes	1	1	2	2

**Table 2:** Fault modes one-hot encoding.

Fault Modes	One-Hot Encoding
Faultless	[1,0,0]
HPC single fault	[0,1,0]
HPC&Fan mixed fault	[0,0,1]

**Table 3:** The specific meanings of 21 sensors.

Symbols	Meanings	Numerical Units
T24	Total temperature at LPC outlet	°R
T30	Total temperature at HPC outlet	°R
T50	Total temperature at LPT outlet	°R
P30	Total pressure at HPC outlet	psia
Nf	Physical fan speed	rpm
Nc	Physical core speed	rpm
Ps30	Static pressure at HPC outlet	psia
Phi	Ratio of fuel flow to Ps30	psia/rpm
NRf	Corrected fan speed	rpm
NRc	Corrected core speed	rpm
BPR	Bypass Ratio	/
htBleed	Bleed Enthalpy	/
W31	HPT coolant bleed	lmb/s
W32	LPT coolant bleed	lmb/s

**Table 4:** The data size after data preprocessing.

Dataset	Initial Data Size	After Denoising	Time Window	Stride	Final Data Size
FD001	(20,631, 26)	(20,631, 14)	30	1	(17,731, 30, 14)
FD003	(24,720, 26)	(24,720, 14)	30	1	(21,820, 30, 14)

**Table 5:** Number of samples for 3 fault modes.

Fault Modes	Number of Samples
Faultless	14,668
HPC single fault	12,402
HPC&Fan mixed fault	12,481

**Table 6:** Comparison results of different models.

Models	Average Accuracy (%)	Macro-Precision (%)	Macro-Recall (%)	F1-Score (%)
SVM	85.37	85.83	85.59	85.71
RF	92.16	92.67	92.09	92.39
FNN	85.70	86.22	85.86	86.04
1DCNN	98.98	98.99	98.99	98.99
RNN	86.67	87.02	86.87	86.95
LSTM	95.40	95.54	95.44	95.49
BiLSTM	97.01	97.05	97.08	97.06
**1DCNN-BiLSTM with CBAM (proposed)**	**99.21**	**99.25**	**99.20**	**99.22**

**Table 7:** Comparison results of ablation experiments.

Models	Average Accuracy (%)	Macro-Precision (%)	Macro-Recall (%)	F1-Score (%)
1DCNN-LSTM	98.51	98.55	98.54	98.54
1DCNN-BiLSTM	99.18	99.19	99.20	99.20
1DCNN-LSTM with CBAM	99.06	99.09	99.06	99.07
1DCNN with CBAM	99.16	99.19	99.15	99.16
**1DCNN-BiLSTM with CBAM (proposed)**	**99.21**	**99.25**	**99.20**	**99.22**

## Data Availability

The study data is supported by NASA Ames Research Center, Moffett Field, CA. Available: https://ti.arc.nasa.gov/tech/dash/groups/pcoe/prognostic-data-repository/, accessed on 1 January 2024.

## Abbreviations

The following abbreviations are used in this manuscript:

CNN	Convolutional Neural Networks
1DCNN	One-Dimensional Convolutional Neural Networks
RNN	Recurrent Neural Networks
LSTM	Long Short-Term Memory
BiLSTM	Bidirectional LSTM
CBAM	Convolutional Block Attention Module
SVM	Support Vector Machine
RF	Random Forest
FNN	Fully Connected Neural Network
RUL	Remaining Useful Life
RtF	Run to failure
